# Dietary patterns, breakfast consumption, meals with family and associations with common mental disorders in adolescents: a school-based cross-sectional study

**DOI:** 10.1186/s12889-022-13367-7

**Published:** 2022-05-16

**Authors:** Lucia Helena Almeida Gratão, Milene Cristine Pessoa, Thales Philipe Rodrigues da Silva, Luana Lara Rocha, Monique Louise Cassimiro Inácio, Tatiana Resende Prado Rangel de Oliveira, Cristiane de Freitas Cunha, Larissa Loures Mendes

**Affiliations:** 1 School of Medicine, Pediatrics Department, Universidade Federal de Minas Gerais, Belo Horizonte, Minas Gerais, Brazil; 2 School of Nursing, Universidade Federal de Minas Gerais, Minas Gerais, Brazil; 3 School of Medicine, Departament of Preventive and Social Medicine, Universidade Federal de Minas Gerais, Belo Horizonte, Minas Gerais, Brazil; 4 School of Nutrition, Universidade Federal de Ouro Preto, Ouro Preto, Brazil; 5 Nutrition Course, Pontifícia Universidade Católica de Minas Gerais, Minas Gerais, Brazil

**Keywords:** Mental Health, Adolescence, Public Health, Food Habits, Eating Behaviors

## Abstract

**Background:**

Adolescence is a period of transition and vulnerabilities, in which mental illnesses can develop more easily. The objective of this work is to analyze the association of dietary patterns, breakfast consumption, and the practice of having meals accompanied by the family with the presence of Common Mental Disorders in Brazilian adolescents.

**Methods:**

This is a cross-sectional study which analyzed data from 71,553 Brazilian adolescents aged 12–17 years, from the Study of Cardiovascular Risk in Adolescents (Portuguese acronym, “ERICA”). Principal Component Analysis was performed to identify dietary patterns, and Logistic Regression Models were performed to identify the associations between Common Mental Disorders, dietary patterns, and eating practices that are breakfast consumption and practice of having meals accompanied by family.

**Results:**

Two patterns were found, a Healthy Dietary Pattern and an Unhealthy Dietary Pattern. Adolescents classified in the second (OR: 0.79; 95% CI 0.70—0.89) or third (OR: 0.86; 95% CI 0.77—0.96) tercile of the Healthy Dietary Pattern had a lower chance of having Common Mental Disorders. Eating breakfast sometimes (OR: 0.71; 95% CI 0.61—0.83) or almost every day/every day (OR: 0.54; 95% CI 0.47—0.62), and the practice of having the main meals with the family sometimes (OR: 0.69; 95% CI 0.57—0.84) or almost every day/every day (OR: 0.50; 95% CI 0.44–0.58), decreased the chance for Common Mental Disorders.

**Conclusion:**

This study observed that healthy dietary patterns are associated with better mental health in adolescents, thus should be encouraged and promoted.

**Supplementary Information:**

The online version contains supplementary material available at 10.1186/s12889-022-13367-7.

## Background

Common Mental Disorders (CMD) refer to two main categories of diagnoses: depressive and anxiety disorders. In addition, there are non-specific and somatic complaints, which may be associated with CMD [1, 2]. Considering depressive and anxiety disorders in isolation, America is one of the continents with the highest prevalence rate in adolescents, around 5.4% and 7.2%, respectively [3].

Also, half of all conditions related to mental health start around 14 years old, characterizing adolescence as a period of transition and vulnerabilities, where mental illness can more easily develop [4, 5]. Additionally, the number of risk factors to which the adolescent remains exposed is directly associated with impacts on mental health [5]. Moreover, in general, during this period, adolescents gained more autonomy in terms of their food choices and eating behavior [6].

Some publications tried to verify isolated associations between consumption of some groups of food and eating practices with depression and/or anxiety. These reports hypothesized that unhealthy eating patterns could act as risk factors in the development of mental illness [7–9]. However, studies have shown that healthy eating patterns, such as those based on fruits, vegetables, and monounsaturated and unsaturated fatty acids, are associated with the prevention of mental disorders [7, 8, 10–13]. Regardless, these studies were conducted in adults or animals, and there are no robust data on the association with adolescents.

In addition to food consumption, dietary practices have also been the subject of studies that have found an association with adolescent mental health. O'Sullivan et al. [14] and Agathão et al. [15] found associations between healthy eating practices such as eating breakfast and eating meals in the presence of family with better mental health in adolescents.

Despite the published scientific literature, there is a gap concerning the association of food consumption and eating practices with the presence of CMD among Brazilian adolescents. Brazil has particular characteristics regarding food culture, territorial extension and socioeconomic factors; therefore, it is important to know how these associations occur in the country [16].

So, we aimed to investigate the association of dietary patterns, breakfast consumption, and the practice of having the meals with family with the presence of CMD in Brazilian adolescents.

## Methods

### Design and sampling process

This is a cross-sectional study with data from the Study of Cardiovascular Risks in Adolescents (Portuguese acronym: ERICA). It was a cross-sectional, national, school-based report with data collection carried out between the period of March 2013 and December 2014, with a sample composed of adolescents aged between 12 and 17 years of both sexes, enrolled in the last three years of elementary school and three years of high school in Brazilian public and private schools.

The sampled population framework was scaled into 32 geographic strata: each capital of the 27 units of the federation; and five strata comprising municipalities with more than 100,000 inhabitants in each of the country's five macro-regions, totaling 273 eligible Brazilian municipalities. After geographic stratification, schools and classes were selected [17, 18].

The schools were selected in each geographic stratum with probability proportional to size (PPS). The size measure of each school was set equal to the ratio between the number of students in its eligible classes and the distance from the State capital. The PPS selection was performed in each geographic stratum after sorting the school records by situation (urban or rural areas) and the school governance (private or public). In the second stage, three classes in each sampled school were selected with equal probabilities during field work. In each selected class, all students were invited to participate. [17, 18]

Detailed information about the sample definition, the sampling process, the research protocol, the participant selection, and the data collection has been published in previous reports [17–19].

### Dependent variable

To build the CMD variable, the Goldberg General Health Questionnaire (GHQ-12) [20] was used, validated for use in adolescents [21]. The GHQ-12 is a widely used self-completed instrument and is known to be a reliable measure of mental health. The GHQ-12 helps to track psychiatric disorders in the community and non-psychiatric clinical settings from an index generated from the individuals' responses [22].

For the screening among the adolescents in this report, the binary system with a cutoff point of five was considered, that is, the presence of CMD was considered when at least 5 of the 12 items were answered with one of the last two options of the questionnaire ("a little more than normal" or "much more than normal"). This cutoff point has a sensitivity of 86.7%, specificity of 88.9%, a positive predictive value of 71.2%, and ROC curve area (Receiver Operating Characteristics) of 0.94 [23].

### Independent variables

#### Dietary patterns

For the construction of the dietary pattern, this report has used food frequently consumed by adolescents. The 24-h recall was the method used to collect data about the dietary food intake of the adolescents participating in the study. This method was applied through face-to-face interviews conducted by trained researchers. A multiple-pass method [24] has been chosen for the interview technique. This technique consists of a guided interview divided into five stages. This interview method intends to reduce food intake underreporting, through the instrument REC24h-ERICA [25].

The software *Brasil-Nutri* [26] was used to record food consumption data. This software contained a list of 1,626 food items from the database regarding the acquisition of food and beverages from the 2002–2003 household budget survey carried out by the Brazilian Institute of Geography and Statistics (“Instituto Brasileiro de Geografia e Estatísticas (IBGE)” in Portuguese), [27] developed by the Ministry of Health in partnership with the Institute of Social Medicine. The database used in the National Dietary Survey was developed by IBGE in 2008–2009 [28].

After converting the weight of the food items into grams, [28] the dataset was linked to a nutritional composition table [28] to calculate the energy consumption of each adolescent. The foods were classified based on the degree of processing, as indicated by the NOVA food classification system [29]. This classification system categorizes all foods into the following 4 groups, according to the nature, extent, and purpose of the industrial processes they undergo: unprocessed and minimally processed food, processed culinary ingredients, processed food, and ultra-processed food. [29] The culinary preparations were disaggregated and their ingredients classified into their respective groups. The food was categorized by 2 independent researchers and discrepancies, if any, were resolved by an expert researcher.

For this study, with the aim of investigating Dietary Patterns among adolescents, was calculated using the Principal Component Analysis (PCA). For the calculation of the Dietary Pattern, only the groups were considered: unprocessed or minimally processed, and ultra-processed foods. This choice was based on the Food Guide for the Brazilian Population (“Guia Alimentar para a População Brasileira” in Portuguese) [29], which recommends that a healthy diet should consist mostly of fresh and minimally processed foods and contain as little ultra-processed food as possible [29]. The ultra-processed foods included in the PCA were: sweetened beverages, packaged snacks, candies, ultra-processed high carbohydrate foods, ultra-processed meat products, cookies (biscuits), milk drinks and dairy products. The unprocessed or minimally processed foods included in the PCA were: vegetables, fruits, cereals, eggs and meat. The foods that were considered in each subgroup are listed in the Supplementary Material.

For this study, beverages that did not specify whether or not they contained added sugar were included in the "Sweetened Beverages" group. Other beverages, as in natura fruit juice, were not considered for this study. This decision was based on studies that showed that eating whole fruit is different from drinking a portion of juice, in nutritional terms [30].

After this process, the kilocalories were calculated. To obtain closer analyzes of the consumption of adolescents, the outliers were removed. Outliers were considered and, consequently, excluded from the present report those adolescents who presented food intake below 500 kcal/day or above 6,000 kcal/day [31].

#### Eating practices

The following variables that refer to eating practices were considered for this report: the practice of having main meals with the family and breakfast consumption. The variable “practice of having the meals with family” was constructed from the grouping of two variables, namely, “Does your father (or stepfather) or your mother (or stepmother) or guardian have lunch with you?” and “Does your father (or stepfather) or mother (or stepmother) or guardian have dinner with you?”. The categories available for the adolescents' response in each question were: (1) my parents or guardians never or almost never have lunch/dinner with me; (2) my parents or guardians have lunch/dinner with me sometimes; (3) my parents or guardians have lunch/dinner with me almost every day; (4) my parents or guardians have lunch/dinner with me every day. In this study we have chosen to merge categories 3 and 4, being the categories of analysis: “never or almost never”, “sometimes” and “almost every day or every day".

The variable “breakfast consumption” had as an answer option in the adolescent's questionnaire: (1) have no breakfast; (2) have breakfast sometimes; (3) have breakfast almost every day; (4) have breakfast every day. The variable was recategorized with the union of the alternatives 3 e 4, being the categories of analysis: “does not consume”, “sometimes” and “almost every day or every day".

### Adjusted variables

The adjusted variables were identified from a theoric model and selected with the aid of a Directed Acyclic Graph (DAG) built in the Dagitty (http://www.dagitty.net/) [32] (Supplementary Material). The minimal sufficient adjustment sets for estimating the total effect of Eating Practices, Dietary Patterns on CMD were age, lives with parents, physical activities, screen time, sex, sleep time, socioeconomic factors and type of school (administrative dependence).

The age of the adolescents was categorized into three age groups: 12–13 years old, 14–15 years old and 16–17 years old. Type of school could be public administration and private administration. As for gender, the alternatives in the student's questionnaire were: female and male.

The variable living with parents has the following categories: lives with both parents, live only with mother or only father, and does not live with either parent.

The categorization of the time of weekly physical activity level practice was performed according to the cutoff points proposed by the National Adolescent Health Survey (Portuguese acronym: PeNSE) [33], in which adolescents who accumulated 300 min or more of physical activity per week were considered physically "active", “insufficiently active 1” those between 1 to 149 min per week, “insufficiently active 2” those who practiced any Physical activity level from 150 to 299 min per week. Students who did not practice any Physical activity level in the week before the interview were considered “inactive”. The questionnaire used by ERICA to assess the practice of physical activity by adolescents was the Physical Activity Questionnaire for Adolescents (QAFA), validated by Farias Junior et al. [34].

The socioeconomic status of adolescents was calculated using the Principal Component Analysis (PCA), to get a Pattern of Socioeconomic Indicators (PSI) (Supplementary Material) with the variables described by Ribeiro et al. [35] and Erwling and Barros [36]. The variables considered were: “number of residents per room”, “employees in the residence”, “number of bathrooms” and “number of refrigerators”. The PSI obtained was characterized by the presence of employees, lower number of residents per room, higher number of bathrooms and higher number of refrigerators (Supplementary Material).

The schools considered in this study were: public and private. To obtain the mean of sleep time, the weighted mean was calculated between the time in hours of sleep usually practiced during weekdays and weekend days, separately. Those individuals who reported a practice of sleeping less than 4 h and more than 14 h were excluded, according to Borges [37]. The daily screen time was classified as greater than three hours a day and less than or equal to three hours a day [38].

### Statistical analysis

The descriptive analysis included the calculation of absolute and relative frequencies for categorical variables, in addition to measures of central tendency and dispersion when the variables were numerical. The chi-square test was performed to compare the proportions between the variables. When the variable had more than 2 categories, the Bonferroni correction was applied to avoid type I errors from multiple comparisons.

To identify the adolescents' dietary patterns, Principal Component Analysis (PCA) was performed, which is a descriptive analytical method that condenses the information contained in the observed variables into a smaller number of variables, with minimal loss of information. For the performance of the PCA, were added all unprocessed or minimally processed, and ultra-processed foods. The Kaiser-Mayer-Olkin (KMO) was estimated as a measure of the adequacy of the PCA, with values between 0.5 and 1.0 considered acceptable for this index. Subsequently, components with Eigen Values > 1.0, defined according to the screen plot graph (Supplementary Material), were extracted from the PCA. The structure of the components was obtained from the indicators that had factor loads greater than > 0.3 or less than -0.3, with a variable being generated in units of points for each consumption pattern. Thus, the following groups were considered: fruits, vegetables, legumes, cereals, meats (unprocessed or minimally processed foods) and sweetened beverages, candies, packaged snacks, cookies (biscuits) (ultra-processed foods). For each pattern obtained in the PCA, a categorical variable was created. The obtained values were distributed in terciles, originating three categories.

To verify the magnitude of the association of dietary patterns, breakfast consumption, and the practice of having meals accompanied by the family with the presence of CMD, using the Odds Ratio (OR) and its 95% confidence intervals (95% CI), the binary logistic regression models was adopted.

The bivariate analysis was performed using simple logistic regression models, with the variable "CMD" as dependent and the variables "dietary patterns", "breakfast consumption" and "practice of having main meals accompanied by parents or guardians" an explanatory.

All analyses adopted 5% significance level. It is noteworthy, that because the Study of Cardiovascular Risks in Adolescents (ERICA) data come from a complex sample, the survey command “svy:” was applied in all statistical analyzes performed in the statistical program Stata software version 14.0 [39].

### Ethical aspects

This report was approved by the Research Ethics Committee of the Institute for Collective Health Studies (“Instituto de Estudos de Saúde Coletiva” in Portuguese) of Federal University of Rio de Janeiro (Portuguese acronym: IESC/UFRJ) which belongs to the report’s central coordination (IESC/UFRJ – Approval nº 45/2008) and of each State. Informed consents were obtained from all subjects, parent and their legal guardian(s). The authors confirm that all methods were performed in accordance with the Declaration of Helsinki.

## Results

### Characteristics of adolescents

This report has evaluated 71,553 Brazilian adolescents. Of those, 50.21% were male, 35.10% with age between 12 to 13 years old, 57.27% lives with their both parents and, 68.21% having the meals with family almost every day or every day and 48.42% has the practice of having the breakfast almost every day or every day (Table 1).

**Table 1:** Characterization of Brazilian adolescents with Common Mental Disorders from the Study of Cardiovascular Risk in Adolescents (ERICA), (*n* = 71,553)

	**Common Mental Disorders**			
**Variable**	**Global** **(%)**	**No** **(%)**	**Yes (%)**	***p* -valueª**
**Sex**				
Female	49.79	46.05	67.92	** < 0.001**
Male	50.21	53.95	32.08	
**Age (Years old)**				
12–13^A^	35.10	36.49	28.39	** < 0.001**
14–15^B^	34.99	34.77	36.06	
16–17^C^	29.90	28.74	35.55	
**Pattern of Socioeconomic Indicators**^b^				
Tercile 1	46.26	46.08	47.11	0.28
Tercile 2	35.04	35.32	33.68	
Tercile 3	18.70	18.60	19.21	
**Type of school**				
Public	83.61	83.70	83.19	0.40
Private	16.39	16.30	16.81	
**Physical activity level**				
Inactive (0 min/week)^A^	16.72	15.59	22.20	** < 0.001**
Insufficiently active 1 (< 150 min/week)^B^	14.19	14.21	14.13	
Insufficiently active 1 (< 300 min/week)^B^	14.05	14.50	11.88	
Active (≥ 300 min/week)^B^	55.04	55.71	51.79	
**Screen time (hours/day)**				
> 3 h a day	58.15	59.41	51.97	** < 0.001**
≤ 3 h a day	41.85	40.59	48.03	
**Live with parents**				
Both parents^A^	57.27	58.48	51.39	** < 0.001**
Only with mother or only with father^B^	36.85	36.08	40.61	
Neither parent^C^	5.88	05.44	7.99	
**Breakfast consumption**				
Does not consume^A^	21.82	19.74	31.91	
Sometimes^B^	29.76	29.39	31.54	** < 0.001**
Almost every day/every day^C^	48.42	50.87	36.55	
**Practice of having the meals with family**				
Never or almost never^A^	8.67	7.33	15.37	** < 0.001**
Sometimes^B^	23.13	22.31	27.34	
Almost every day/every day^C^	68.21	70.36	57.39	

ª Chi-square test; **bold:** statistically significant; Equal letters mean similarity between the proportions of the groups, and different letters mean difference between the proportions of the groups

^b^ The pattern of socioeconomic indicators was characterized by a higher number of employees at home, a lower number of residents per room, a higher number of bathrooms at home and a higher number of refrigerators at home (Supplementary Material)

Regarding the level of PA, 16.72% of adolescents were classified as inactive, and 14.19% as insufficiently active 1, that is, with physical activity level for less than 150 min per day (Table 1). Furthermore, 58.15% of the students stayed longer than recommended for their age group in front of screens (Table1). According to the classification of the Brazilian Society of Pediatrics (“Sociedade Brasileira de Pediatria” in Portuguese) [26], with a daily time of fewer than 3 h a day, for adolescents.

### Characteristics of adolescents with CMD

It has been observed that, in this sample, girls (67.92%) had more CMD than boys (32.08%) (< 0.001), and among adolescents between the ages of 14 and 15 years old (36.06%) (< 0.001) (Table1).

Those with presence of CMD, compared to those without presence of CMD, 22.20% were inactive in physical activities (< 0.001). As for the practice of having the main meals with the family, the percentage of adolescents who belongs to the category “almost every day or every day” was mostly higher among those who did not have CMD (70.36%) compared to those with CMD (57.39%) (< 0.001). Additionally, those with presence of CMD consumed less frequently the breakfast (36.55%) (< 0.001), respectively (Table 1).

### Dietary patterns

The results for the identification of dietary patterns of adolescents, presented in Table 2, identified two patterns, with a contribution of 28.78% of explained accumulated variation. The KMO index, the factor loadings and Bartlett’s sphericity test were satisfactory. Pattern 1 was characterized by higher consumption of vegetables, legumes, cereals, and meats, called in this report the Healthy Dietary Pattern (HDP), and pattern 2 was characterized by higher consumption of sweetened beverages, candies, chips, and stuffed sweet cookies and lower consumption of fresh fruit, and was called an Unhealthy Dietary Pattern (UDP) (Table 2).

**Table 2:** Factor loads of the Principal Component Analysis of Dietary Patterns of Brazilian adolescents, (*n* = 71,553)

Indicator	**Healthy Dietary Pattern**	**Unhealthy Dietary Pattern**	KMO^a^
Sweetened Beverages	0.2080	**0.5152**	0.5591
Candies	-0.0217	**0.3227**	0.5114
Packaged snacks	-0.03337	**0.3399**	0.5494
Cookies (biscuits)	0.0572	**0.4301**	0.5259
Vegetables	**0.3171**	-0.1973	0.5654
Fruits	-0.1848	**-0.5046**	0.5675
Legumes	**0.4684**	0.0005	0.5751
Cereals	**0.5563**	-0.1067	0.5864
Eggs and Meats	**0.5369**	-0.1581	0.5896
*Eigenvalue*	*1.43952*	*1.15046*	
*Explained variance (%)*	*15.99*	*12.78*	
*Cumulative variance explained (%)*	*15.99*	*28.78*	
*Overall*			*0.5756*
***Bartlett’s sphericity test***			***p* < *0.001***

^a^ Kaiser–Meyer–Olkin. **Bold**: statistically significant

### Association between dietary pattern, eating practices and CMD

The variables associated with the presence of CMD in the multiple logistic regression models are shown in Table 3. Based on the results, we identified that adolescents classified in the second (OR: 0.79; 95% CI 0.70 -0.89) or third (OR: 0.86; 95% CI 0.77—0.96) tercile of the HDP, that is, those who consumed more kilocalories from healthy foods (vegetables, legumes, cereals, and meats) had a lower chance of having CMD.

**Table 3:** Crude and adjusted logistic regression analysis: probability of Common Mental Disorders in Brazilian adolescents

Variable	Common Mental Disorders	
	Crude OR (CI95%)	Adjusted OR (CI95%)^a^
**HDP**		
First tercile	(Ref.)	(Ref.)
Second tercile	0.81 (0.72—0.90)***	0.79 (0.70—0.89)**
Third tercile	0.71 (0.65—0.78)***	0.86 (0.77—0.96)*
**UDP**		
First tercile	(Ref.)	(Ref.)
Second tercile	1.12 (0.98—1.27)	1.00 (0.86—1.15)
Third tercile	1.30 (1.13—1.49)***	1.17 (0.99—1.38)
**Breakfast consumption**		
Does not consume	(Ref.)	(Ref.)
Sometimes	0.66 (0.59—0.75)***	0.71 (0.61—0.83)***
Almost every day/every day	0.45 (0.40—0.50)***	0.54 (0.47—0.62)***
**Practice of having meals with family**		
Never or almost never	(Ref.)	(Ref.)
Sometimes	0.58 (0.50—0.67)***	0.69 (0.57—0.84)***
Almost every day/every day	0.38 (0.34—0.43)***	0.50 (0.44—0.58)***

*OR* Odds Ratio, *CI* Confidence Interval, *UDP* Unhealthy Dietary Pattern, *HDP* Healthy Dietary Pattern, **bold** statistically significant

^*^ *p* < 0.05

^**^ *p* < 0.01

^***^ *p* < 0.001

^**a**^ Adjusted by Physical activity level, socio-economic status, gender, age, screen time, type of school, sleep time, lives with parents

As for eating practices, eating breakfast sometimes (OR: 0.71; 95% CI 0.61—0.83) or almost every day/every day (OR: 0.54; 95% CI 0.47—0.62), and the practice of having the main meals with the family sometimes (OR: 0.69; 95% CI 0.57—0.84) or almost every day/every day (OR: 0.50; 95% CI 0.44—0.58), decreased the chance for CMD in Brazilian adolescents belonging to this report (Table 3).

## Discussion

This cross-sectional and representative report for the Brazilian population showed that having a HDP, characterized by the consumption of vegetables, legumes, cereals, and meat, is associated with a lower chance of CMD in adolescents. Moreover, eating practices of breakfast consumption and the practice of having the main meals with the family regularly are associated with a lower chance of CMD.

Reports with non-Brazilian adolescents have found similar results when evaluating the association between consumption of healthy diets and the diagnosis of depression and anxiety. The adoption of healthy eating is associated with better mental health in adolescents [40]. Renzaho, Kumanyika & Tucker [41], in a cross-sectional work with Australian children and adolescents aged between 4 and 12 years old, that showed that the consumption of fruits and vegetables was associated with protection against emotional problems.

In this context, in addition to acting in the prevention of Chronic Non-Communicable Diseases (NCDs), the adoption of a healthy diet can contribute to reducing the chances of CMD. Therefore, encouraging practices that promote healthy and adequate eating can be useful in preventing these disorders in adolescents. In Brazil, the National School Food Program (Portuguese acronym: PNAE) seeks to encourage and guarantee the right to adequate and healthy food for children and adolescents enrolled in public schools [42].

The Food Guide for the Brazilian Population [29], of the Ministry of Health, encourages fresh and minimally processed food choices, based on a healthy daily diet, and advises against the consumption of ultra-processed foods in all age groups. The Brazilian Food Guide, as well as those from other countries as Canada and England, encourage healthy eating practices, such as eating breakfast and eating meals together [30, 43, 44].

In the present study, we found that healthy eating practices, such as eating breakfast and having meals with the family were inversely associated with the presence of CMD in adolescents. Kipping breakfast was associated with increased the risks of stress and depressive mood in Korean adolescents [45]. Fulkerson et al. [46], investigating adolescents in Minnesota (USA), found that depressive symptoms were negatively associated with healthy practices, as eating the main meals (breakfast, lunch and dinner). However, these studies were centered local and is not representative for theirs countries or others locations.

Breakfast consumption is also associated with positive health outcomes, such as prevention of overweight and other NCDs [47], improved cognition, and adequate school performance in children and adolescents [48, 49]. Thus, the planning and consumption of healthy foods in this meal is also a protection strategy against CMD [14].

Moreover, the presence of the family during meals is crucial for the construction of healthy eating practices, so much that the higher frequency of family meals is associated with fewer depressive symptoms and fewer emotional difficulties in adolescents [50]. Agathão et al. [15], in a longitudinal report, found that Regular meals with the family were a protective factor for the mental health of Brazilian adolescents aged between 12 and 17 years. Furthermore, the chance of having depression can be up to 4.5 times greater in adolescents who do not have the practice of having meals with the family (frequency less than or equal to once a week) [51].

It is understood that family meals are opportunities in which family members can connect and strengthen their bonds [15, 52]. Therefore, having meals with the family can be considered a protective factor for the presence of CMD in adolescents. In addition, the presence of parents at meals is positively associated with higher consumption of fruits, vegetables, and dairy products by adolescents [53]. Consequently, strategies that encourage families to eat together should be promoted.

This report has limitations, such as the cross-sectional design, which no causality between nutritional factors and mental health can be established, and the variables used in the composition of dietary patterns, which were constructed from a single 24-h food recall, which may not accurately characterize the food intake of adolescents. However, the strengths of this report should be considered, such as its originality, as we did not find, within our searches, articles that discuss the association of eating patterns, eating practices, and CMD.

## Conclusion

The results found in this report demonstrate that having a HDP, eating breakfast and having the practice of having the main meals with family regularly reduce the chance of CMD.

Thus, we recommend that initiatives to promote proper and healthy eating should be strengthened and encouraged. With the results of this article it was possible to realize that these actions can prevent not only diseases related to chronic noncommunicable diseases, but also mental illnesses in adolescents.

Action projects, such as facilitating access and availability of fresh and minimally processed foods, taxation of ultra-processed food products, regulation of the sale of unhealthy foods in school environments, and nutrition education with the families of adolescents are initiatives that have been discussed in developing countries and need incentives to be implemented.

In addition, multicenter longitudinal studies should be conducted to investigate causes and effects of common mental disorders in adolescents.

## Supplementary Information

**Additional file 1. **Supplementary Material.

## Data Availability

The datasets used and/or analysed during the current study are available from the Study of Cardiovascular Risks in Adolescents (Portuguese acronym: ERICA) Comitee on reasonable request.

## Abbreviations

CMD: Common Mental disorders
GHQ-12: Goldberg General Health Questionnaire
PCA: Principal Component Analysis
ROC: Receiver Operating Characteristics
IBGE: Instituto Brasileiro de Geografia e Estatística
DAG: Directed Acyclic Graph
WHO: World Health Organization
KMO: Kaiser Meyer Olkin
UDP: Unhealth Dietary Patterns
HDP: Health Dietary Patterns
PPS: Probability proportional Size
PSI: Pattern of Socioeconomic Indicators
QAFA: Physical Activity Questionnaire for Adolescents
OR: Odds Ratio
NCDs: Chronic Non-Communicable Diseases
PA: Physical Activity
