# Supplementary material for: Dietary patterns, breakfast consumption, meals with family and associations with common mental disorders in adolescents: a school-based cross-sectional study
Source: BMC Public Health. 2022 May 16;22:980. doi: 10.1186/s12889-022-13367-7 (PMC9109384; doi:10.1186/s12889-022-13367-7)
Supplement: Supplementary file 1 — Additional file 1. Supplementary Material. [file 12889_2022_13367_MOESM1_ESM.docx]

**Supplementary Material**

1. **Description of the food subgroups included in Independent Variable – Dietary Pattern**

Chart 1. Foods included in the groups used for the principal component analysis.

| **Food Subgroup** | **Included Foods¹** |
| --- | --- |
| **Ultra-processed food** | |
| Sweetened Beverages | Soft drinks with added sugar, fresh fruit juices with added sugar, powdered or industrially produced juices, chocolate drinks with added sugar, coffee with added sugar and energy drinks. |
| Packaged snacks | All kinds of packaged snacks with industrial ingredients. |
| Candies | Chewing gum, caramels, lollipops, and other candies produced with industrial ingredients were considered for the construction of the candies group. |
| Cookies (biscuits) | All kinds of cookies with fillings. |
| Milk drinks and dairy products | Milk-based foods: cheese, milk-based cream, yogurt, milk-based reconstitution, foods milk-based with industrial ingredients. |
| Ultra-processed high carbohydrate foods | Mass-produced packaged breads and buns; powdered and packaged “instant” noodles; cakes and cake mixes; and many ready-to-heat products, including prepared pies and  pasta and pizza dishes. |
| Ultra-processed meat products | Sausage, mortadella, ham, salami and any kind of processed meat (pork, beef, chicken, fish, …). |
| **Unprocessed food or minimally processed** | |
| Vegetables | Carrots, potatoes, onions, beets, lettuce and other leafy foods, tomatoes, chayote, cucumbers, among other vegetables that can be acquired in their natural form and have not suffered the addition of industrial ingredients). |
| Fruits | Apple, banana, pear, strawberry, pineapple, peach, grapes and all other fruits that can be acquired in their natural form and have not suffered the addition of industrial ingredients |
| Legumes | All kinds of in natura legumes as beans, lentils, chickpeas, peanuts, peas, soybeans. |
| Cereals | All kinds of in natura cereals as rice, oats, corn, barley, quinoa, amaranth, wheat (pasta and other processed products were not included). |
| Eggs and Meats | All kinds of eggs and meat without addition of industrial ingredients (pork, beef, chicken, fish and others). |

¹Only the foods cited by the teenagers in 24-hour food recall were considered.

1. **Principal Component Analysis of the Pattern of Socioeconomic Indicators**

**Method**

To identify the Pattern of Socioeconomic Indicators (PSI), a Principal Component Analysis (PCA) was performed, which is an exploratory analytical method that condenses the information contained in the observed variables into a smaller number of variables, with minimal loss of information.

To perform the PCA of the PSI, the variables were considered: employees in the residence, number of residents per room, number of bathrooms in the residence and number of refrigerators in the residence. The Kaiser-Meyer-Olkin (KMO) was estimated as a measure of adequacy of the PCA, with values between 0.5 and 1.0 considered acceptable for this index.

Subsequently, the components with eigenvalue greater than 1.0, defined according to the screen plot graph, were extracted from the PCA. The structure of the components was obtained by the indicators that presented factor loads greater than 0.3 or less than -0.3, being generated a variable in score units for the socioeconomic patterns, named according to the indicators retained. For the pattern, a categorical variable was created from the tercile values of the distribution of scores of these patterns.

**Table 1.** Factor loads of the first component of the Principal Component Analysis of the Pattern of Socioeconomic Indicators of Brazilian adolescents included in the ERICA study. Brazil, 2013-2014.

| **Indicators** | **Pattern of Socioeconomic Indicators** | **KMO^¥^** | |
| --- | --- | --- | --- |
| Employees in the residence | 0.4668 | 0.6162 |  |
| Number of residents per room | -0.4315 | 0.5213 |  |
| Number of bathrooms | 0.6417 | 0.5365 |  |
| Number of refrigerators | 0.4291 | 0.5601 |  |
| *Eigenvalue* | *1.44892* | *.* |  |
| *Explained variance (%)* | *36.22* | *.* |  |
| *Overall* | *.* | *0.5505* |  |
| ^¥^Kaiser-Meyer-Olkin | | |  |

1. **Directed Acyclic Graph (DAG)**


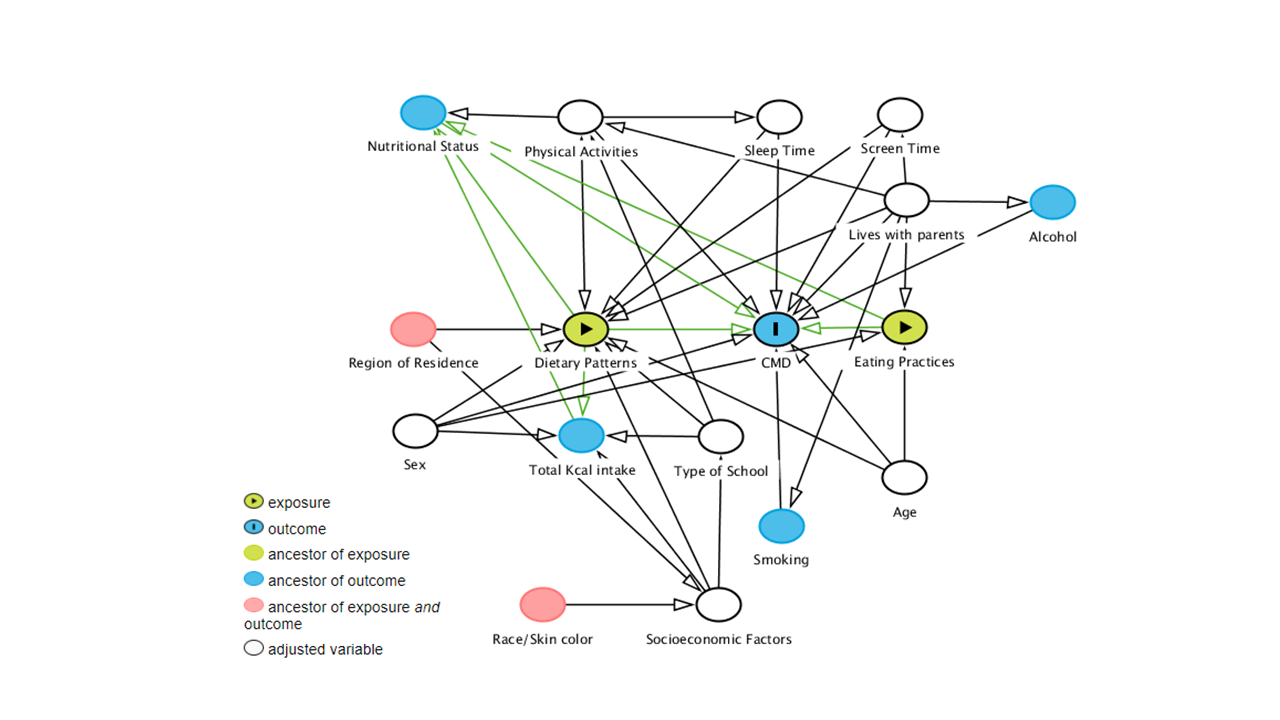


Figure 1. Directed Acyclic Graph (DAG): Dietary Patters, Eating Practices (exposure) and Common Mental Disorders (CMD) (outcome). Minimal sufficient adjustment sets for estimating the total effect of Eating Practices, Dietary Patterns on CMD were Age, Lives with parents, Physical Activities, Screen Time, Sex, Sleep Time, Socioeconomic Factors, Type of School.

1. **Scree plot of eigenvalues after PCA of Dietary Patterns**

Figure 2. Scree plot of eigenvalues after PCA of Dietary Patterns.

**References**

Erwling F, Barros ADJ. How changes in asset ownership affect the national economic indicator in 10 years? Revista Saude Publica. 2017. doi: 10.1590/S1518-8787.201705100651.

Ribeiro, I.B.S. et al. Common mental disorders and socioeconomic status in adolescents of ERICA. Revista de Saúde Pública. 2020, 54(4).

Textor J. et al. Robust causal inference using directed acyclic graphs: the R package 'dagitty'. International Journal of Epidemiology 2016; 45(6):1887-1894.
